# Design and validation of a novel dosimetry phantom for motion management audits

**DOI:** 10.1002/acm2.70091

**Published:** 2025-04-09

**Authors:** Alex Burton, Sabeena Beveridge, Nicholas Hardcastle, Silvio Malfitana, Janaka Madamperuma, Rick Franich

**Affiliations:** ^1^ Australian Clinical Dosimetry Service (ACDS) Australian Radiation Protection and Nuclear Safety Agency (ARPANSA) Yallambie Victoria Australia; ^2^ Department of Physical Sciences Peter MacCallum Cancer Centre Melbourne Victoria Australia; ^3^ Sir Peter MacCallum Department of Oncology University of Melbourne Melbourne Victoria Australia; ^4^ School of Science RMIT University Melbourne Victoria Australia; ^5^ Centre for Medical Radiation Physics University of Wollongong wollongong New South Wales Australia

**Keywords:** dosimetry audit, motion management, radiochromic film

## Abstract

**Background:**

We present a novel phantom design for conducting end‐to‐end dosimetry audits for respiratory motion management of two anatomical treatment sites. The design enables radiochromic film measurements of the dose administered to the target throughout the respiratory cycle (motion‐included) and the dose delivered to the time‐averaged motion of the phantom (motion‐excluded) to be conducted simultaneously.

**Purpose:**

To demonstrate the phantom's utility in a dosimetry audit and capacity to detect errors by quantifying spatial and dosimetric reproducibility.

**Methods:**

Spatial and dosimetric reproducibility was quantified by repeat exposures using a simple lateral beam. Five exposures per measurement configuration were used. In each series of five measurements, the median film was used as the series reference to quantify the reproducibility of the remaining “test films.” Spatial reproducibility was quantified by comparing the position of isodose lines in two axes on the test films back to the series reference. Dosimetric reproducibility was quantified using gamma comparison between each test film and the series reference. Proof‐of‐concept of the motion‐excluded measurement capability was also established by comparing all films to treatment planning system (TPS) calculated dose distributions.

**Results:**

Spatial reproducibility was better than 1 mm on all assessed metrics across all measurements. Film‐to‐film local gamma passing rates at 3%/0.6 mm were above 90% for all measurements. Film‐to‐TPS global gamma passing rates at 3%/1 mm were >95% in the motion‐excluded measurement series’, but <80% in the motion‐included series, highlighting the utility of the motion‐excluded measurements.

**Conclusions:**

Measurements were highly reproducible and of sufficient accuracy/reproducibility to facilitate multiple avenues of analysis in a prospective dosimetry audit. Motion‐excluded measurements were directly comparable to the TPS dose distribution. Motion‐included measurements may yield more clinically‐relevant information about the actual dose administered to the target. This promises greater sensitivity to motion management‐related errors, detectable in the setting of a dosimetry audit for motion management.

## 1 INTRODUCTION

End‐to‐end dosimetry audits are performed to measure the accuracy with which the audited facility can deliver a prescribed dose to a known treatment target.^1^, ^2^ This requires a comparison between the planned and measured dose distributions, typically in an anthropomorphic phantom used to represent a specific treatment site such as the brain or thorax. This can become complicated in the context of motion management, where the phantom is irradiated while it moves in a periodic manner representing respiratory motion. Planned doses, however, are not temporally‐resolved or time‐integrated, and therefore do not include the dose‐blurring effects that will be present in the measurement. This also means the planned dose is, by design, not a precise calculation of the actual dose received by the target as it moves through the respiratory cycle. The planned coverage of the target's motion envelope is rather an estimate of what is achieved. As such, an error free delivery cannot necessarily be verified by direct comparison of the delivered dose to the dose distribution in the treatment plan.

Several solutions have been proposed to this problem. One is to simply perform the measurement without any motion; however, this is suboptimal because it does not properly simulate key sources of uncertainty and potential errors that may arise from using motion management techniques.^3^ A second option is to use relaxed comparison criteria to reduce sensitivity to the motion‐induced dose differences, however, this simultaneously reduces sensitivity to error‐induced dose differences as well.^4^, ^5^, ^6^ A third, highly novel solution was proposed, which enabled a motion‐exclusive measurement to be performed while the phantom was moving.^7^ This was achieved using 3D printing to suspend a sheet of Gafchromic™ EBT4 film (Ashland Global Specialty Chemicals Inc, Covington, USA) statically through the center of the measurement insert, while the two halves of the insert were driven synchronously in the superior‐inferior direction on either side of it. This approach enabled a direct comparison between the measured and planned doses. However, this simply enabled the validation of an inherently imprecise dose calculation. We have recently shown how the sensitivity of motion‐inclusive measurements to errors may be improved (relative to whole film analysis) by using region‐of‐interest (ROI) gamma analysis.^6^ Therefore, there is merit in performing motion‐inclusive measurements as a more direct measurement of the dose received by the treatment target. Other novel audit phantoms developed for lung stereotactic body radiation therapy (SBRT) have utilized motion‐inclusive measurements to assess the accuracy of both passive and active motion management techniques.^8^, ^9^

We present a novel phantom design enabling motion‐inclusive and ‐exclusive measurements to be conducted simultaneously. This design was created to efficiently facilitate two avenues of analysis in a prospective dosimetry audit under development at the ACDS. In this work we conducted a series of tests to demonstrate the accuracy and reproducibility of the phantom with the aim of demonstrating its appropriateness for use in a prospective national‐scale motion management dosimetry audit.

## 2 MATERIALS AND METHODS

### 2.1 Phantom design

**FIGURE 1:**
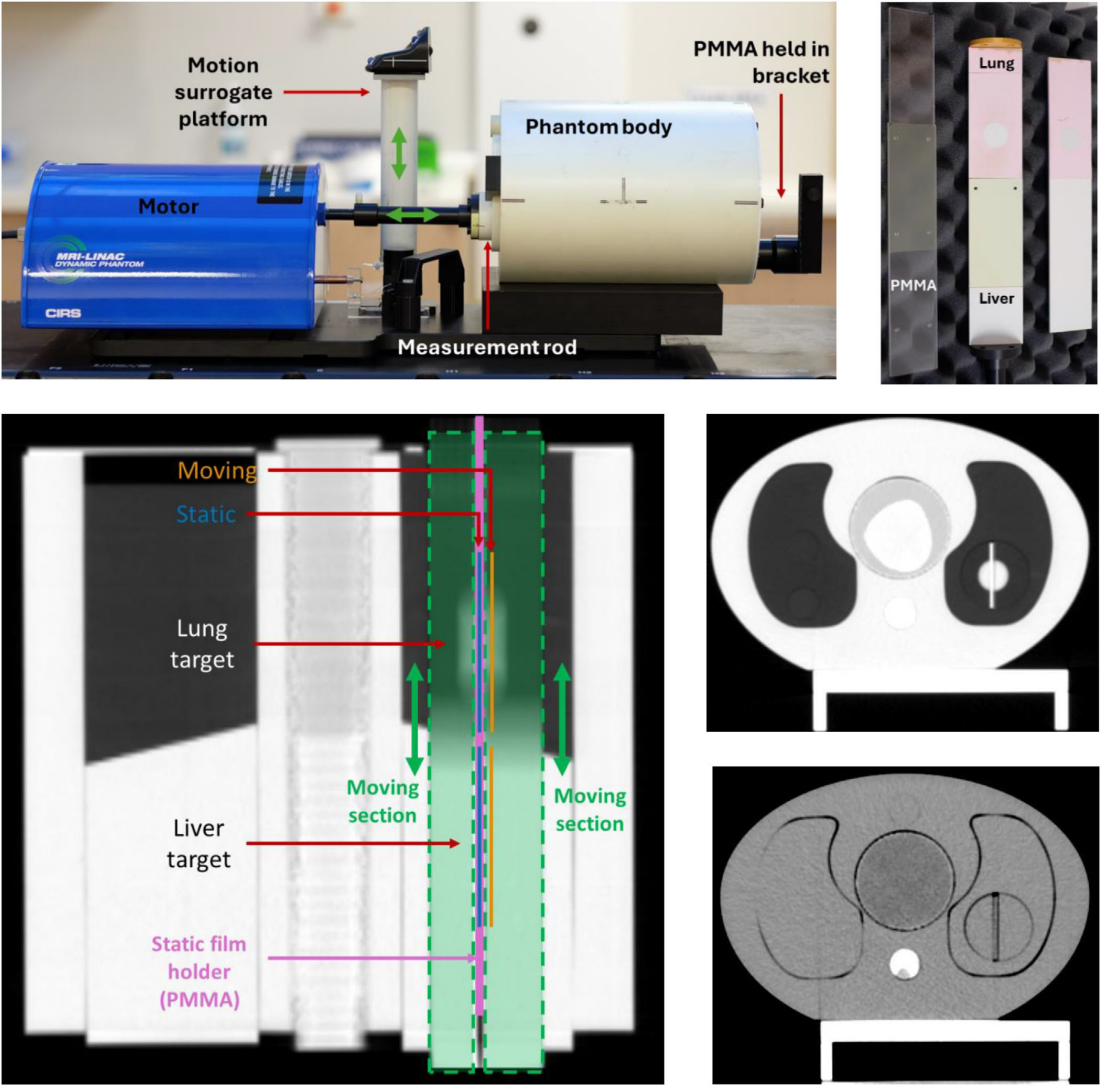
Top left—picture of the assembled phantom mounted on the CIRS 008Z motion platform. Top right—disassembled measurement rod showing films in‐situ in both static and moving measurement locations for the lung treatment site. The film in the latter location is covered by a thin layer of CIRS media. Bottom left—coronal slice of a CT showing the internal location of the measurement positions. Blue lines show the positions of the static films, and orange lines show the moving films. Green shaded regions show the two cylinder halves, separated by the PMMA holder in pink. Bottom right—axial CT slices at the level of the lung target (top) and liver target (bottom). PMMA, polymethyl methacrylate.

The phantom consists of a custom CIRS SBRT phantom body (Sun Nuclear, Melbourne, USA) mounted to the CIRS 008Z MRgRT motion platform (Figure 1). The phantom body was constructed of CIRS Plastic Water DT, with removable “lungs” constructed of either Plastic Water DT or Lung Inhale materials. The removable lungs were modified to create a “liver” treatment site out of Plastic Water DT in the inferior portion of the phantom and a “lung” treatment site out of Lung Inhale in the superior portion of the phantom. These two sections meet at an approximate 15° angle in the coronal plane at the midpoint of the phantom body to represent a diaphragm. A 55 mm diameter cylindrical cavity was machined out of the left removable lung/liver insert for its entire length to allow passage of a cylindrical measurement rod to mate to the 008Z motor and provide motion in the superior‐inferior direction. The measurement rod was machined out of the same materials as the surrounding lung/liver insert and was split along its longitudinal axis in the sagittal plane into two unequal sections. A 25 mm diameter spherical “lesion” made from Plastic Water DT was embedded in the lung section, 45 mm superior to the diaphragm. There was no visible “lesion” in the liver section to simulate the lack of target contrast typically seen in non‐contrast CT. The internal face of the larger cylinder section was recessed by 2 mm to allow the placement of 125 × 54 mm pieces of Gafchromic™ film, covered by an approximate 1.5 mm thick sheet of CIRS material in each of the treatment sites. This enabled motion‐inclusive measurements to be conducted in the central sagittal plane in each of the treatment sites, separated by 90 mm in the superior‐inferior direction. This was termed the *moving* measurement location. The large and small cylinder sections were separated by a 2.5 mm gap to allow insertion of a second *static* measurement location, constructed of two 1 mm thick sheets of polymethyl methacrylate (PMMA). The PMMA extended past the end of the measurement rod and was mounted to the stationary phantom body using a 3D printed bracket. It contained locator pegs to align a 122 × 53 mm piece of Gafchromic™ film parallel and 3 mm medial to the moving measurement location. With the motor active, the two cylinder sections move synchronously in the superior‐inferior direction, while the PMMA sheets and the film between them remain static. This design therefore facilitated the simultaneous acquisition of motion‐inclusive and ‐exclusive measurements (in the moving and static measurement positions, respectively) for both the lung and liver treatment sites.

All machining was performed on commercial milling machines rated to less than 0.1 mm precision, resulting in minimal air gaps. CIRS materials were used due to their radiological equivalence to biological materials, and to enable the future use of previously defined dose‐to‐medium correction factors.^10^, ^11^, ^12^ The static measurement plane was constructed from PMMA rather than CIRS materials due to concerns over the structural integrity and friction between the static and moving parts.

### 2.2 Experimental design

**FIGURE 2:**
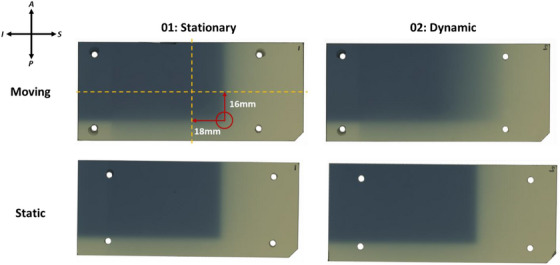
Raw scans of the films from deliveries 1 and 2 demonstrating the difference between the stationary and dynamic measurement series (respectively), as well as the isocentre position (red circle) relative to the film centre (yellow dashed lines). The orientation of the films in the phantom is shown in the top left. The moving film in delivery 2 (top right) is blurred in the superior‐inferior direction as a result of the applied motion, while the static film irradiated at the same time has no blurring in this axis (bottom right).

In this experiment, we use the terms static and moving to refer to the two different measurement positions in the phantom. The terms stationary and dynamic refer to the status of the motor. The phantom was irradiated with a 6 MV beam from a Varian True Beam linear accelerator (Varian Medical Systems, Palo Alto, USA) with standard Millennium 120 multileaf collimators (MLC). The isocenter was placed midway between the static and moving measurement planes in the medio‐lateral direction, 18 mm inferior and 16 mm posterior to the center of the film at each of the treatment sites (Figure 2). A lateral 10 × 10 cm beam (gantry 90°, collimator 0°) with two non‐divergent jaws was used to irradiate the phantom with 1000 monitor units (MU). Simple beam geometry was used to enable direct measurement of spatial and dosimetric reproducibility with reference to the fixed field borders and to minimize uncertainties arising from beam modeling or delivery errors in more complicated irradiations. Both static and moving measurement locations were loaded with laser‐cut pieces of Gafchromic™ EBT4 film in every beam delivery. Each beam delivery therefore resulted in two irradiated films. The phantom was irradiated under both stationary and dynamic conditions. In the stationary measurements, the motor was switched off, and no motion was applied. In the dynamic measurements, the phantom was programmed to move with a linear sawtooth motion profile (amplitude: ± 20 mm, period: 5 s). Five stationary and five dynamic measurements were performed at each of the lung and liver treatment sites. This resulted in 20 paired results and 40 irradiated films in total: eight series of five repeat measurements, with one series for each combination of measurement location (moving or static), treatment site (lung or liver), and motor state (stationary or dynamic). Figure 2 shows the raw scans of the films from deliveries 1 (stationary) and 2 (dynamic) in both static and moving measurement locations, with a mark‐up showing the isocenter position relative to the center of the films. During the experiment, beam deliveries were numbered from 1 to 20, alternating between stationary measurements (all odd‐numbered beams) and dynamic measurements (even numbers). The first 10 beams were delivered to the lung treatment site; the second 10 to the liver treatment site.

All films were scanned at 120 dpi in transmission mode to TIF format using an Epson Expression 12000XL flat‐bed scanner (Seiko, Suwa, Japan). Conversion to RGB dose maps in TIF format was performed using a 12‐point calibration curve from the same film lot ranging up to 25 Gy irradiated on the same day as the experiments, using in‐house software written in Python v3.11^13^, ^14^ Calibration doses were delivered using a 6MV flattened beam under reference conditions in solid water using a Farmer‐type PTW 30013 ionization chamber (PTW, Freiburg, Germany) directly tracible to the Primary Standards Dosimetry Lab in Australia. Calibration and experimental films were irradiated on the same day and scanned 6 days later to ensure consistent darkening (uncertainty <0.5%). All experimental films were scanned simultaneously with the same two calibration films of known dose, which were used to correct for scan‐to‐scan variation (uncertainty <0.5%).

The ACDS film process uses the net optical density of the calibration films to fit a third‐degree polynomial function to generate calibration curves in each color channel. A pre‐scan of the calibration films (prior to irradiation) was performed to calculate the initial optical densities for the calibration films. Quality assurance tests are performed throughout the entire film process and the ACDS estimates their film dosimetry uncertainty to be within 2.3% as described in Beveridge et al.^13^

Rigid translations and rotations were applied to the scanned films to align the locator peg holes to a common position in the image for analysis. Films in the lung treatment site (deliveries 1–10) were registered to film 1, and films from the liver treatment site^11^, ^12^, ^13^, ^14^, ^15^, ^16^, ^17^, ^18^, ^19^, ^20^ were registered to film 11. Different registrations were performed to absorb any setup uncertainties because the phantom was set up separately for each treatment site. The RGB dose maps were converted to grayscale images and de‐noised using a 3 × 3 pixel median filter for further analysis using scipy v1.13.0^15^ and PyMedPhys v0.40.0^16^ for gamma analysis.

### 2.3 Dosimetric analysis

First, the “in‐field” dose was defined for the lung and liver treatment sites. This was determined by masking the films based on a dose threshold, and then taking the mean dose within this ROI. The dose threshold was iteratively increased from 6.5 Gy, until the difference between the median stationary and dynamic films in each series was less than 0.05 Gy. This process was done dosimetrically (rather than spatially) to exclude the impact of any spatial variance, in particular, in the motion‐inclusive measurements where the penumbra was broadened in the motion axis. The threshold in the lung treatment site was 8.2 Gy, and the threshold in the liver site was 7.2 Gy, resulting in average ROI areas of 2700 mm^2^ and 2250 mm^2^ respectively. Example ROIs are depicted in Appendix A.1. These dose thresholds were used as the normalisation values in later gamma analyses.

The mean dose in the ROIs defined by these dose masks was used for two applications. First, it was used to define the 100% in‐plane isodose in each individual measurement, used in later analyses. Secondly, the film with the median ROI dose in each series of 5 measurements was used to define the series reference film, with the remaining 4 referred to as test films.

Dosimetric reproducibility was assessed using gamma analysis. In each series, test films were compared to the series reference using dose difference criteria ranging from 1% to 4%, with a fixed distance criteria of 0.6 mm. The small distance criteria was selected to reflect the small spatial uncertainty of the film registration process and was set at 0.6 mm to acknowledge the use of the 3 pixel de‐noising (median) filter. Local gamma with a 5% low dose threshold was used to scrutinize the entire dose distribution, including the penumbra/out‐of‐field regions. Additionally, global gamma with a 20% low‐dose threshold was used to constrain analysis to the high‐dose region more typical of the analysis used in dosimetry audits.^5^, ^17^

The linearity of the results in the static and moving locations was quantified using Pearson's correlation for the 1%/0.6 mm gamma results. This was performed to examine the relationship between results in the pairs of simultaneously irradiated films and attempt to expose any systematic errors affecting both.

### 2.4 Spatial analysis

Spatial reproducibility was assessed by comparing the position of the 5 Gy isodose line and penumbra width in both the motion (superior‐inferior SI) and non‐motion (anterior‐posterior AP) axes in each of the test films to the series reference. The 5 Gy isodose line was a measurement of the jaw position, representing the 50% isodose relative to the nominal global dose maximum of 10 Gy (given the 1 cGy/MU at the depth of maximum dose calibration). The penumbra width was measured from the 80% to the 20% isodose line, relative to the 100% in‐plane isodose determined earlier.

To predict the penumbra width in the motion axis of the moving measurement location, the dynamic dose profile was modeled by convolving the stationary measurements with a 1D averaging kernel. Each 1D dose profile in the motion axis was convolved with a kernel defined by:

(1) $$\frac{1}{n}\;\left[\begin{array}{c} 1_{i}\\ \text{…}\\ 1_{n} \end{array}\right]$$

where *n* represents the width of the blurred region. This was 40 mm, corresponding to the peak‐to‐peak amplitude of the phantom. Using this kernel, each position in the dose profile was weighted by an even fraction of the total motion to provide linear blurring corresponding to the sawtooth motion profile. This was iteratively applied to each row in the motion axis for the dynamic measurements. The convolved stationary measurements were then compared to the dynamic measurements to quantify how accurately the motion was captured.

### 2.5 Comparison to TPS

A 10 phase‐binned 4DCT of the phantom was acquired using a Philips CT scanner and the Varian Respiratory Gating for Scanners (RGSC) device. Dose was calculated on the average intensity projection (AIP) in the Eclipse v16.1 treatment planning system (TPS) using AcurosXB dose‐to‐medium. Dose planes corresponding to both the moving and static measurement locations were exported for comparison to the measurements. Gamma passing rates at 2%/1, 3%/1 and 4%/1 mm (all global gamma, 20%TH) were reported for the moving series of measurements in both treatment sites.

## 3 RESULTS

### 3.1 Dosimetric analysis

The results of the in‐field dose masking are shown in Table 1. The mean dose shown in Table 1 was used to define the 100% in‐plane isodose for each individual film.

**TABLE 1:** Mean and standard deviation of dose within an ROI defined by the 8.2 Gy isodose line for the lung site, and 7.2 Gy isodose line for the liver site.

	Lung site			Liver site	
Meas. location	Moving	Static		Moving	Static
Delivery no.	Mean ± 1SD [Gy]	Mean ± 1SD [Gy]	Delivery No.	Mean ± 1SD [Gy]	Mean ± 1SD [Gy]
1	9.10 ± 0.37	9.07 ± 0.40	11	8.58 ± 0.33	8.47 ± 0.38
2	9.06 ± 0.36	9.03 ± 0.35	12	8.53 ± 0.38	8.42 ± 0.33
3	9.18 ± 0.43	9.10 ± 0.43	13	8.59 ± 0.38	8.48 ± 0.40
4	9.14 ± 0.44	8.99 ± 0.42	14	8.54 ± 0.38	8.45 ± 0.35
5	9.19 ± 0.42	9.00 ± 0.36	15	8.64 ± 0.35	8.39 ± 0.34
6	9.22 ± 0.45	9.08 ± 0.37	16	8.59 ± 0.39	8.41 ± 0.34
7	9.14 ± 0.40	9.10 ± 0.43	17	8.54 ± 0.41	8.40 ± 0.34
8	9.12 ± 0.44	9.02 ± 0.31	18	8.53 ± 0.39	8.32 ± 0.32
9	9.22 ± 0.43	9.09 ± 0.39	19	8.61 ± 0.36	8.33 ± 0.36
10	9.06 ± 0.39	8.99 ± 0.33	20	8.55 ± 0.39	8.41 ± 0.32

Abbreviations: ROI, region‐of‐interest.

**FIGURE 3:**
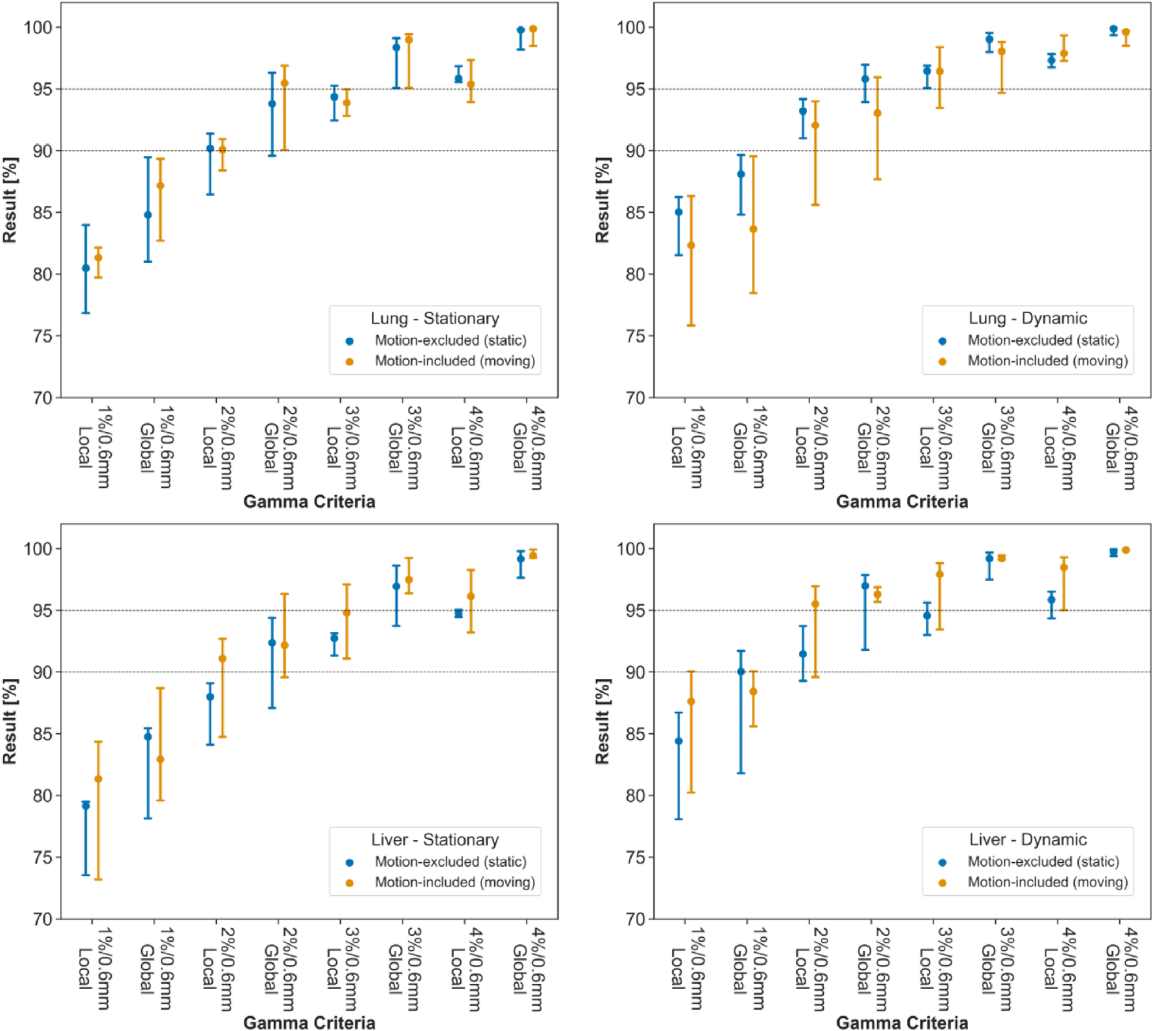
Median and range of gamma passing rates in each series, comparing the test films to the series reference across all tested gamma criteria.

The results of the gamma analysis are shown in Figure 3. At 3%/0.6 mm, median passing rates in all series were >95% in the global gamma comparison, and all test results were >90% in the local gamma comparison. The largest spread in results was in the moving measurement location for the dynamic lung measurements (Figure 1b), but the range in passing rates was <5% once the dose difference criteria was above 2%. Passing rates at 1%/0.6 mm for the static and moving locations correlated poorly with one another (global gamma: *ρ* = 0.022, *p* = 0.47, local gamma: *ρ *= 0.28, *p* = 0.17). Some example gamma maps are provided in Appendix A.2.

### 3.2 Spatial analysis

**FIGURE 4:**
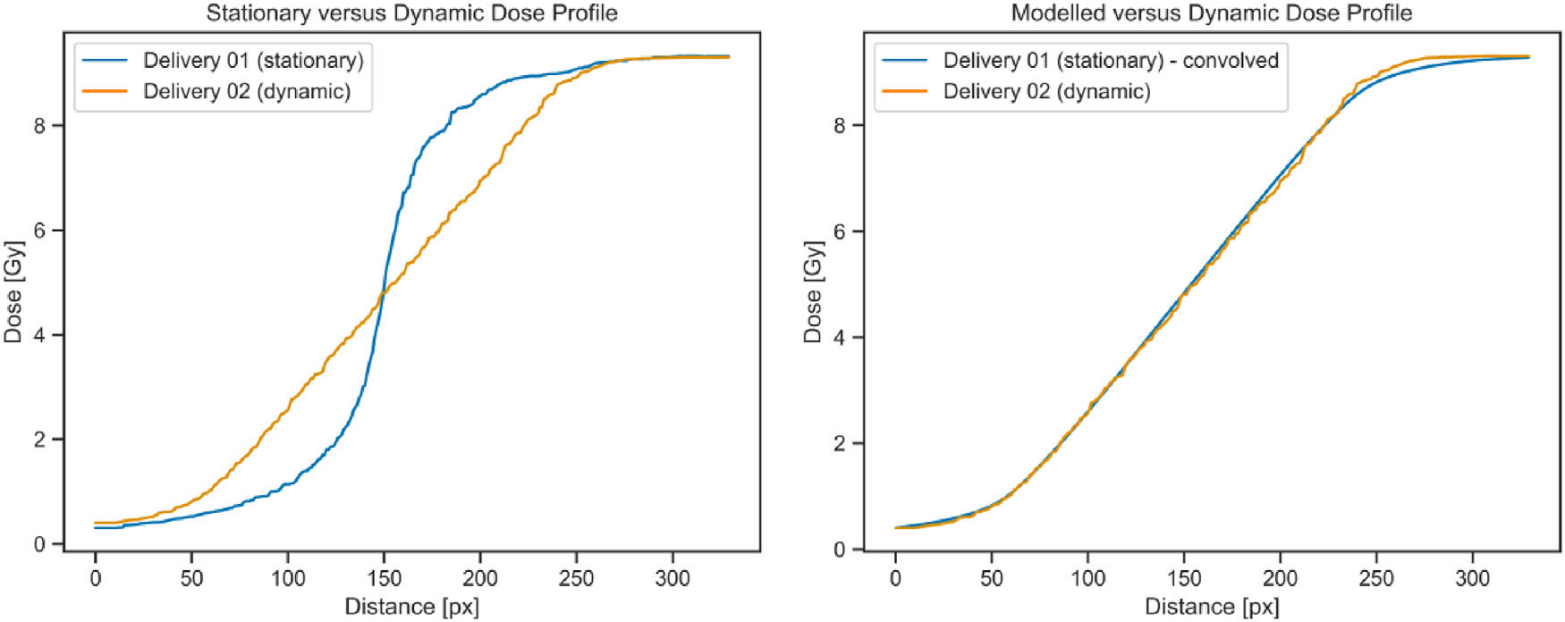
Example of the process used to validate the motion capture in the dynamic series. The plots on the left show the actual measured dose profiles in the SI axis for stationary (delivery 01 ‐ blue) and dynamic (delivery 02 ‐ orange) test films. In the plot on the right, the stationary dose profile has been convolved with a linear blurring kernel.

Both 5 Gy isodose position and penumbra width were highly reproducible, with no deviations from the series reference larger than 0.5 mm observed in any series (Table 2). The dynamic and stationary series were compared using the reference in each series only. The 5 Gy isodose position in both axes and the penumbra width in the non‐motion axis were unaffected by the motion for both lung and liver treatment sites (Table 3). For the static measurement position, the penumbra in the motion axis was not affected by the addition of motion. For the moving measurement position, however, the penumbra increased by approximately 11 mm (Table 3). When the dynamic series were compared to the convolved‐stationary series (see Figure 4 for an example), the measured 5 Gy isodose position and penumbra width in the motion axis were within 1 mm of the modeled prediction (Table 3).

**TABLE 2:** Results of the spatial reproducibility assessment, showing the median [range] differences (in mm) to the series reference.

		Lung moving	Lung static	Liver moving	Liver static
*SI 5 Gy isodose position*	*Stationary*	0.3 [0.1–0.4]	0.3 [0.2‐0.3]	−0.2 [−0.3–0]	0.1 [0–0.1]
	*Dynamic*	0.2 [0–0.5]	0 [‐0.3‐0.2]	0.3 [0.2–0.5]	−0.1 [−0.4–0.3]
*SI penumbra*	*Stationary*	−0.1 [−0.3–0]	0.1 [0.1‐0.3]	0 [0–0.1]	0.1 [0–0.2]
	*Dynamic*	0.3 [0.1–0.5]	0.1 [0.1‐0.2]	0 [−0.2–0.4]	−0.1 [−0.5–0.3]
*AP 5 Gy isodose position*	*Stationary*	0 [−0.1–0.1]	−0.1 [−0.1–0]	0.2 [−0.1–0.3]	0 [−0.2–0.5]
	*Dynamic*	0.2 [0–0.3]	0.1 [−0.1–0.2]	0.2 [0–0.5]	0.2 [0–0.3]
*AP penumbra*	*Stationary*	0.1 [−0.1‐0.5]	0.2 [0–0.2]	0 [0–0.1]	0 [−0.2–0.4]
	*Dynamic*	−0.1 [−0.2–0]	0.1 [−0.1–0.1]	−0.1 [0.1–0]	0.1 [0–0.3]

*Note*: Positive values indicate the median was larger than the series reference.

**TABLE 3:** Difference (dynamic minus stationary) in spatial parameters between series references in the same measurement position/treatment site (in mm).

	Lung moving	Lung static	Liver moving	Liver static
*SI 5 Gy isodose position*	−0.2	0.1	0.3	0
*SI penumbra width*	10.9	−0.1	11.1	0
^a^ *Convolved SI 5 Gy isodose position*	−0.3	–	−0.6	–
^a^ *Convolved SI penumbra width*	−0.3	–	−0.7	–
*AP 5 Gy isodose position*	−0.2	−0.1	−0.2	0.1
*AP penumbra width*	0.1	−0.1	0.1	0

^a^ Comparison between the actual dynamic measurements and the modeled dynamic measurements produced via convolution.

### 3.3 Comparison to TPS

**FIGURE 5:**
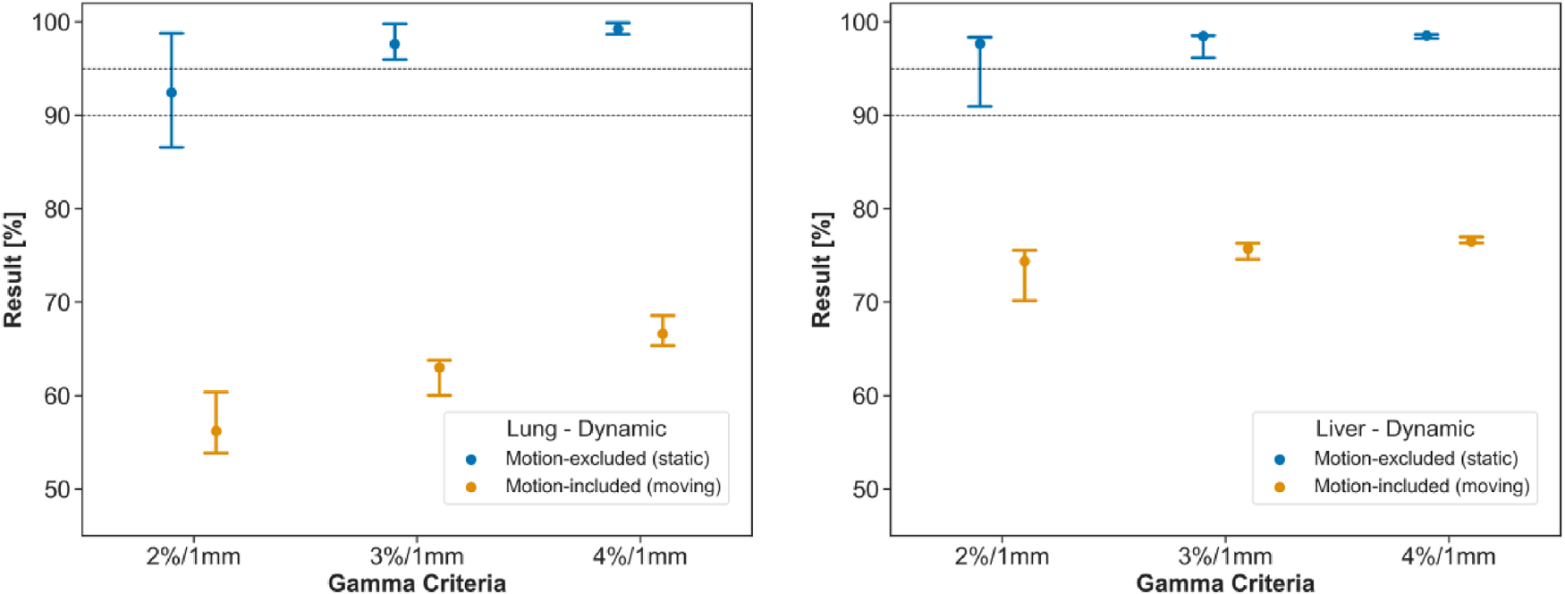
Median and range of gamma passing rates in dynamic measurement series in lung (left) and liver (right) treatment sites comparing the measurements to the planned dose.

**FIGURE 6:**
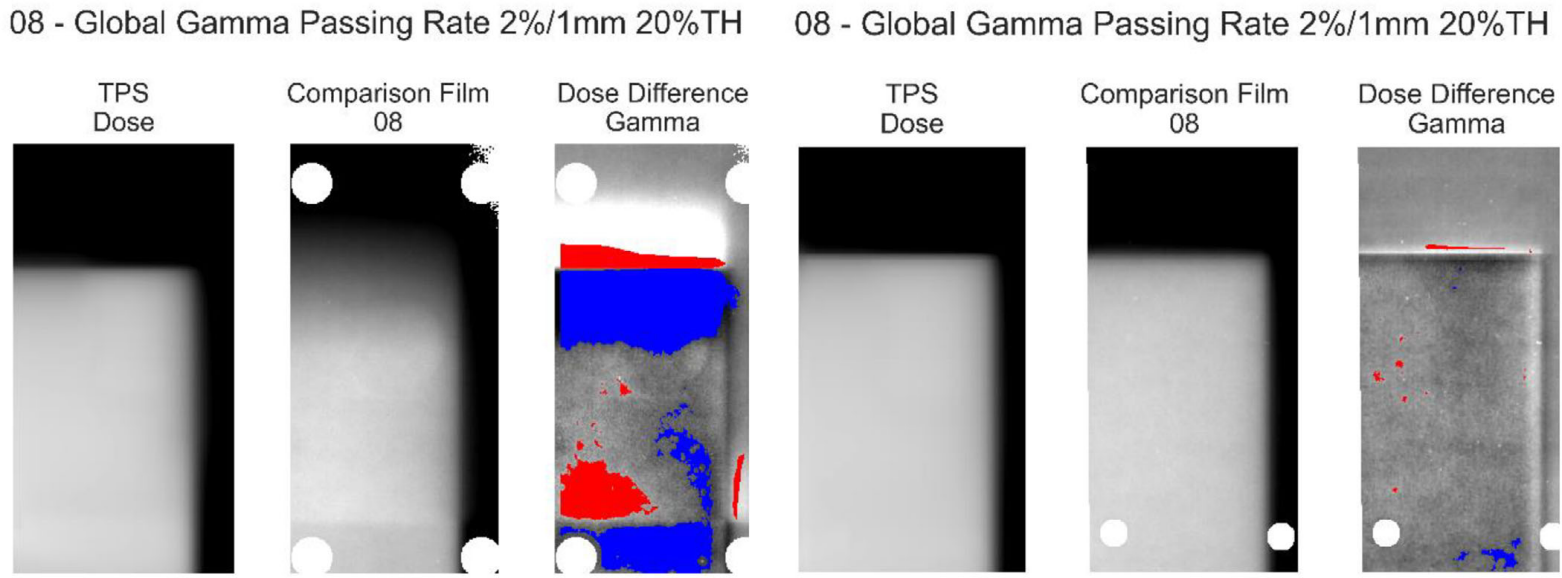
Example of a pair of simultaneously irradiated films compared to their respective TPS dose planes. Left is the moving measurement location, and right is the static location. Both show the global gamma map at 2%/1 mm overlaid on the dose difference (TPS minus measured) map. Blue/red pixels correspond to lower/higher measured dose compared to TPS, respectively. TPS, treatment planning system.

The results of the gamma comparison to the TPS are shown in Figure 5. At 3%/1 mm, all passing rates were >95% in the static measurement position, while passing rates in the moving measurement position were substantially degraded. At 4%/1 mm assessment criteria, the range in gamma passing rates for repeat measurements was reduced to <3.5% in all series. Figure 6 provides an example of a pair of gamma maps in the static and moving measurement positions, irradiated at the same time, demonstrating that the failing regions correspond directly to the motion‐affected areas of the film.

## 4 DISCUSSION

Several bespoke phantoms have been developed for the purpose of film‐based end‐to‐end dosimetry audits in the context of lung SBRT. The IROC thorax phantom is the most prominent example, having been in use for more than 10 years. Other phantoms have been developed for smaller‐scaled dosimetry audits with a focus on anatomically realistic appearance and respiratory characteristics.^8^, ^9^, ^18^ The design presented in this work is distinguished from previous phantoms by the inclusion of a liver treatment site in the same phantom assembly, having large film measurement areas (>50 mm in both axes) and, most critically, by the ability to simultaneously perform motion‐inclusive and ‐exclusive measurements. This design offers the advantage of enabling multiple avenues of analysis from a single irradiation. The motion‐excluded measurement allows direct comparison to the entire equivalent planned dose plane,^7^ while the motion‐included measurement enables the dose delivered to the GTV to be more directly quantified.^8^, ^19^ Having both arms of analysis was a key feature of the prospective audit to ensure its sensitivity to the broadest range of error types, not limited to those impacting the dose distribution in the center of the field (away from the motion‐induced dose blurring effects at the field borders). One limitation of the motion‐excluded measurement plane was that it restricted the applied motion to only the superior‐inferior direction, which may not mimic all clinical scenarios. It also required the inclusion of non‐biological equivalent materials (PMMA) in the assembly, which is suboptimal for dosimetry. However, the benefits of having the two arms of analysis from a single exposure were deemed to outweigh these drawbacks. The corrections required due to the PMMA are the subject of further work from this group.

The aim of this work was to validate this novel phantom design and assess its suitability for use in a prospective dosimetry audit for motion management to be implemented on a national scale through the ACDS. The relative complexity of the design presented challenges for reproducibility of results and could potentially result in high measurement uncertainties in practice. Spatially, the performance of the phantom was very good. We confirmed that the film in the static measurement position (in PMMA) remains static, even when the rest of the measurement cylinder is moving around it. In addition, we confirmed that the moving section of the phantom was moving as expected given the applied motion. This speaks to the high precision with which the custom parts were fabricated.

The dosimetric reproducibility as assessed by simple ROI metrics (Table 1) was also suitably high. The range in the in‐field mean dose was at its largest in the moving (lung) measurement location and was only 0.16 Gy (1.8%) across all 10 measurements (grouping both moving and static). These results were similar in magnitude to the variation in films irradiated under reference conditions,^14^ and so it seems that the complex design of the phantom has similarly not substantially increased our measurement uncertainty. The results of the gamma analysis also support this conclusion. The measurements were minimally post‐processed, with only a gentle de‐noising filter applied, and no manual masking of obvious film aberrations such as visible variations in substrate or active layer. Passing rates remained high, despite using intentionally penalizing assessment criteria (i.e.,—setting the normalization dose to a value at approximately 90%–95% of the in‐field maximum and a sub‐mm distance criteria). The resulting gamma maps (see Appendix A.1) did not yield any discernible pattern in the failing pixels, indicating that no systematic errors were introduced. The lack of correlation in passing rates between simultaneously irradiated films also points to an absence of systematic errors.

The comparison to the TPS was included as a proof‐of‐concept of the phantom design, demonstrating the advantage of including a static measurement plane. It also showed how the spatial/dosimetric variation in repeat measurements quantified in the previous tests may translate to variation in audit outcomes. The small variation in outcomes indicated that the phantom can facilitate the use of sufficiently discerning gamma criteria (such as those recommended by the AAPM Task Group 218^20^), and the audit will hence be capable of detecting clinically relevant errors. The passing rates themselves were comparable to those of Retif et al.^7^ (the only other example of a motion‐excluded measurement in the literature), who found an average passing rate of 93.1%, 96.6%, and 97.6% at 2%/1, 3%/1, and 4%/1 mm respectively, for 5 dynamic conformal arc irradiations. The results found in this study may be further improved by additional post‐processing of the film as described in the previous paragraph. In addition, the measurements did not have dose‐to‐medium correction factors applied, which have previously been shown to improve agreement.^11^

We have previously demonstrated how motion‐inclusive measurements may be optimally utilized to detect clinically realistic errors, where gamma passing rates within specific ROIs were shown to correlate with reductions in target coverage.^6^ The availability of two forms of analysis strengthens both the sensitivity and specificity of the dosimetry audit. For example, any suboptimal results in the motion‐excluded measurement position can be meaningfully contextualized by their potential clinical implications, based on the result in the motion‐inclusive measurement position.

## 5 CONCLUSION

We developed a novel phantom capable of performing simultaneous motion‐inclusive and ‐exclusive film measurements. The phantom facilitated measurements of sufficient accuracy and precision to detect clinically relevant errors in the setting of a dosimetry audit. The new phantom, combined with an optimized scoring approach, allows sensitive audit measurements to be conducted without the need to concede loose passing criteria to accommodate the large uncertainties previously experienced using existing approaches.

## AUTHOR CONTRIBUTIONS

**Alex Burton**: Conception; experimental and phantom design; acquisition; data processing; analysis and interpretation; manuscript preparation. **Sabeena Beveridge**: Conception; experimental design; manuscript editing. **Nicholas Hardcastle**: Conception; experimental design; manuscript editing. **Silvio Malfitana**: Phantom design and fabrication; manuscript review. **Janaka Madamperuma**: Phantom design and fabrication; manuscript review. **Rick Franich**: Conception; experimental design; manuscript editing.

## CONFLICT OF INTEREST STATEMENT

N.H. receives research grant funding for work on kidney and prostate SABR and prostate radionuclide therapy. N.H. is a paid consultant of SeeTreat Medical. A.B. and N.H. work for an institution which receives funding from Varian Medical Systems to provide a workshop.

## APPENDIX A

### A.1 Appendix

Example scanned films with showing the masked region used to define the “in‐field” dose shaded in light red. Note the reduction in size of the ROI in the dynamic measurements (relative to the stationary measurements) due to the broadening of the penumbra in the motion axis.

### A.2 Appendix

Example of two pairs of measurements with global gamma maps at 2%/0.6 mm overlaid.
